# Limitations of Bulk Diamond Sensors for Single-Cell Thermometry

**DOI:** 10.3390/s24010200

**Published:** 2023-12-29

**Authors:** Andrea Alessio, Ettore Bernardi, Ekaterina Moreva, Ivo Pietro Degiovanni, Marco Genovese, Marco Truccato

**Affiliations:** 1 Physics Department, University of Turin, Via P. Giuria 1, 10125 Turin, Italy; 2 Istituto Nazionale di Ricerca Metrologica, Strada delle Cacce 91, 10135 Turin, Italy

**Keywords:** bio-sensing, diamond temperature sensors, finite element analysis

## Abstract

The present paper reports on a Finite Element Method (FEM) analysis of the experimental situation corresponding to the measurement of the temperature variation in a single cell plated on bulk diamond by means of optical techniques. Starting from previous experimental results, we have determined—in a uniform power density approximation and under steady-state conditions—the total heat power that has to be dissipated by a single cell plated on a glassy substrate in order to induce the typical maximum temperature increase $\Delta T_{glass}=1$ K. While keeping all of the other parameters constant, the glassy substrate has been replaced by a diamond plate. The FEM analysis shows that, in this case, the maximum temperature increase is expected at the diamond/cell interface and is as small as $\Delta T_{diam}=4.6\times 10^{-4}$ K. We have also calculated the typical decay time in the transient scenario, which resulted in τ≈ 250 μs. By comparing these results with the state-of-the-art sensitivity values, we prove that the potential advantages of a longer coherence time, better spectral properties, and the use of special field alignments do not justify the use of diamond substrates in their bulk form.

## 1. Introduction

Temperature plays a central role in cell metabolism. Temperature acts as a parameter, e.g., regulating the speed of ion channel opening [1], but, in turn, it is also affected by cell activity, e.g., mitochondrial activity induces intracellular temperature variations [2]. Furthermore, an increase in temperature is related to pathological conditions, such as cancerous cells [3], Parkinson’s disease, and Alzheimer’s diseases [4]. Considering this central role, temperature measurement at the intracellular scale gives an essential insight into understanding the cell’s behavior both in normal and pathological conditions, with the perspective of improving the medical treatment of diseases.

From a theoretical point of view, there is still some debate about the presence of a general thermodynamical principle governing biological processes. In particular, the minimum entropy production principle is valid only in the linear response regime [5]. A recent idea, named dissipation-driven adaptation [6], has been proposed that considers far-from-equilibrium conditions, even if some criticisms have already been raised [7]. Measuring the temperature near the cellular organelles can shed light on this topic, giving information about the dissipation rate of specific metabolic processes.

In recent years, an increase in local temperature of up to a few Celsius degrees has been detected as a consequence of calcium stress [8] or of the use of drugs that increase the heat produced during cellular respiration [9,10,11]. The power needed to justify this temperature increase has been calculated to exceed by a few orders of magnitude what is expected from thermodynamic considerations [12,13,14,15]. A way to tackle this problem can be based on the simultaneous measurements of temperature at the intracellular level by using different methods to check for consistency of the different measurement results.

Several intracellular thermometry solutions are reported in the literature, ranging from fluorescent molecular thermometers [16] to quantum dots [17] to rare earth nanoparticles [18]. In particular, color centers in nanodiamonds have been widely used in the past few years for intracellular temperature measurements due to their significant advantages [19,20]. Nitrogen-Vacancy (NV) centers have been used in combination with Optically Detected Magnetic Resonance (ODMR); see [21,22] and the references therein. ODMR consists of the measurement, by optical means, of the resonance frequency of the electronic ground state of the NV centers [23]. Group IV defects in diamond [24], such as Silicon-Vacancy (SiV) [25,26], Germanium-Vacancy (GeV) [27], and Tin-Vacancy (SnV) [28], have been used [29] as thermometers, taking advantage of the temperature-dependent shift in their photoluminescence spectra. In the future, they might be used for intracellular measurement.

In general, nanodiamonds achieve nanometric resolution, are biocompatible [30,31], and can be functionalized [32]. A recent work [33] has experimentally proved, using SiV centers, that a nanoscale heat source leads to a significant temperature gradient at the sub-micrometric scale in an aqueous solution, whereas another very significant contribution has demonstrated the first measurement of temperature effects related to the synaptic activity at the intracellular level, using NV centers [34]. On the other hand, nanodiamonds present a short coherence time [35,36], considering ODMR measurements, and a degree of imperfection that limits spectral properties [37], which is detrimental for any measurement involving photoluminescence spectra. Concerning this last point, the use of diamond crystallites of micrometer size and with a high aspect ratio, called diamond microneedles, is interesting. Indeed, microneedles are enriched with color centers at the synthesis stage, leading to better structural and optical properties compared to nanodiamonds [38].

From the point of view of thermal measurements, both a nanodiamond and a diamond microneedle are much smaller than a cell. So, the temperature of the nanodiamond, or of the diamond microneedle, equilibrates to that of the cell, as demonstrated by Finite Element Analysis (see Supplementary Materials in Ref. [38]), leading to the possibility of sensing the temperature at the nanometric scale.

The possibility of using bulk diamonds instead of nanodiamonds would be interesting. Considering ODMR measurements on NV ensembles, nanodiamonds present a lower sensitivity compared to bulk diamonds, because of their shorter coherence time (as already mentioned), the lower density of their color centers, and the difficulty in taking advantage of the field alignment [39] and laser polarization [40]. On the contrary, a bulk diamond allows ODMR measurements with micrometric spatial resolution on the x-y plane using a focused laser beam and nanometric resolution along the z-direction using a diamond with a thin layer of NV centers [41]. Furthermore, a bulk diamond can be nanostructured to form an array of diamond nanopillars, leading to the possibility of guiding the growth of the cell network [42] and to further improvements in sensitivity due to the pillars acting as optical waveguides [43].

The main problems with measuring intracellular temperature using a bulk diamond are represented by its large mass compared to that of the cell and by its high thermal conductivity. As a result, the cell temperature equilibrates quickly on a short temporal and spatial scale to the bulk temperature, which acts similarly to a thermal bath. This implies, in general, a short acquisition time and a short distance from the cell in order to be able to measure a temperature signal inside the cell.

The aim of this paper is to model and quantify, by means of Finite Element Analysis, the temporal and spatial scale of a thermal signal produced by cellular processes for a cell adhered to a bulk diamond. We consider both the transient and stationary cases, discussing whether it is possible to measure a cellular temperature given the relevant spatio–temporal scale and the state-of-the-art sensitivities of diamond-based probes.

The paper is organized as follows: In the **Materials and Methods** section, the model used for Finite Element Analysis is described, and the thermal parameters for the different components of the model are presented. In the **Results** section, first, in a steady-state scenario, we estimate the total heat power dissipated $P_{diss}$ by a cell adhered to a glass substrate, considering a temperature increase of $\Delta T_{glass}=1$ K, which is typical of recent experiments [9,34]. Then, we calculate the temperature profiles induced by the same power $P_{diss}$ but for a cell adhered to a bulk diamond substrate. We calculate also the typical decay time in the transient scenario. In the **Discussion** section, we examine the possibility of measuring the calculated temperature profiles, considering the state-of-the-art experimental sensitivities. In the **Conclusions** section, we comment on the limitations of the proposed model, and we propose future works.

## 2. Materials and Methods

### Finite Element Analysis

**Figure 1:**
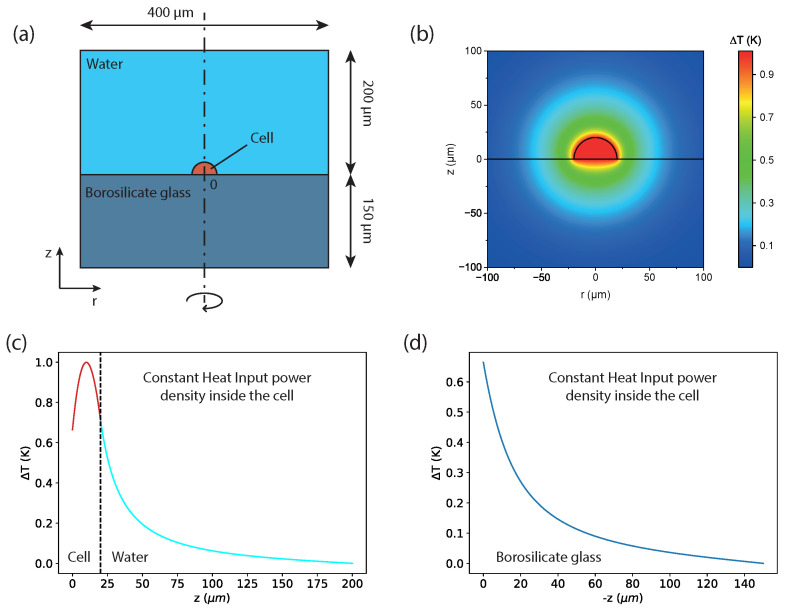
(**a**) Sketch of the model used for the cell–water–borosilicate experimental setup: (i) the cell, colored in orange, modeled as a hemisphere of 40 μm of diameter; (ii) the water environment, colored in blue, above the cell, modeled as a cylinder with a base of 400 μm and a height of 200 μm; and (iii) the borosilicate sample, colored in dark blue below the cell, modeled as a cylinder with a base of 400 μm and a height of 150 μm. The total dissipated heat power integrated over the whole cell is fixed at $P_{diss}=1.04\times 10^{-4}\;W$ and corresponds to a heat power density Q that is constant throughout the cell. The temperature of the boundaries is fixed at 310.15 K (37.00 ${}^{\circ }$C). (**b**) Map of the temperature increase in the z-r plane. (**c**) Temperature increase profile along the positive z-axis, i.e., inside the cell and then in the water, and along negative z (**d**). In (**c**,**d**), the radial position is always r=0 μm. All of the temperatures are represented in terms of their increase ΔT with respect to the temperature of the boundaries (37.00 ${}^{\circ }$C).

**Figure 2:**
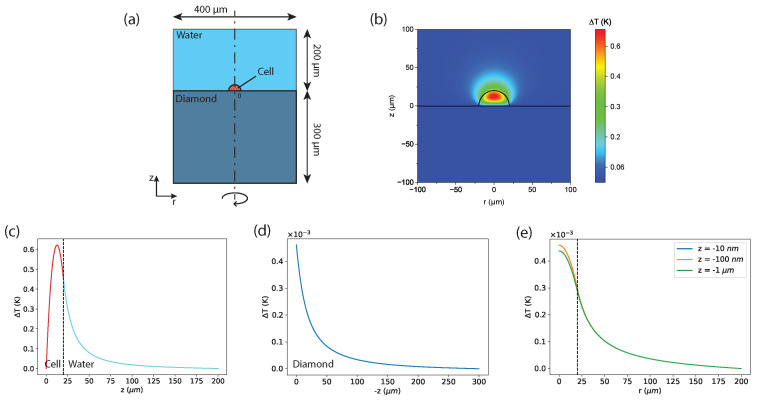
Results of the stationary scenario. (**a**) Sketch of the model using cell–water–diamond: (i) the cell, colored in orange, modeled as a hemisphere of 40 μm of diameter; (ii) the water environment, colored in blue, above the cell, modeled as a cylinder of a base of 400 μm and a height of 200 μm; and (iii) the bulk diamond sample, colored in light blue below the cell, modeled as a cylinder with a base of 400 μm and a height of 300 μm. The origin of the frame of reference is located at the center of the cell hemisphere. The total dissipated heat power integrated over the whole cell is fixed at $P_{diss}=1.04\times 10^{-4}\;W$ and corresponds to a heat power density Q that is constant throughout the cell. The temperature of the boundaries is fixed at 310.15 K (37.00 ${}^{\circ }$C). (**b**) Map of the temperature increase in the z-r plane. (**c**) Temperature increase profile along the positive z-axis, i.e., inside the cell and then in the water, and along negative z (**d**), i.e., inside the diamond. (**c**,**d**) have different scales. In (**c**,**d**), the radial position is always r=0 μm. (**e**) Spatial decay along r of the temperature increase for z = −0.01 μm, z = −0.1 μm, and z = −1 μm, i.e., at 3 different depths inside diamond. All of the panels show the temperature increase with respect that of the boundaries (37.00 ${}^{\circ }$C).

Finite Element Analysis is performed by means of the COMSOL Multiphysics software package, v 5.5 (COMSOL AB: Stockholm, Sweden). A 3D model with cylindrical symmetry is used (see Figure 1a and Figure 2a) and is filled with a mesh of up to 274,525 triangular elements, with a minimum element size of 5 nm. A mesh sensitivity analysis has been carried out and reported in Figure S1 in Supplementary Material. Numerical simulations were carried out on a computer with 88 GB of RAM and two processors with a clock of 2.4 GHz, and the computation times did not exceed 5 min.

For the stationary scenario, the classical time-independent heat equation is implemented [44]:

(1) ∇·(−k∇T)=Q

Dirichlet boundary conditions are imposed by setting the temperature of the external surfaces of the model at the fixed value $T_{boundary}=310.15$ K, i.e., 37.00 ${}^{\circ }$C. The heat source *Q* is defined as a uniform power density generated by the cell. This power density is fine-tuned in order to have a maximum temperature inside the cell of 311.15 K, i.e., a maximum temperature difference of 1 K, when the cell is in contact with a borosilicate glass substrate, as it was experimentally observed in [34] (see Figure 1). A precision of $10^{-5}$ K is necessary for the specification of the boundary conditions due to the minimal temperature differences resulting from the simulations.

The very same power density is then inserted in the stationary simulation with a diamond substrate; see Figure 2.

**Figure 3:**
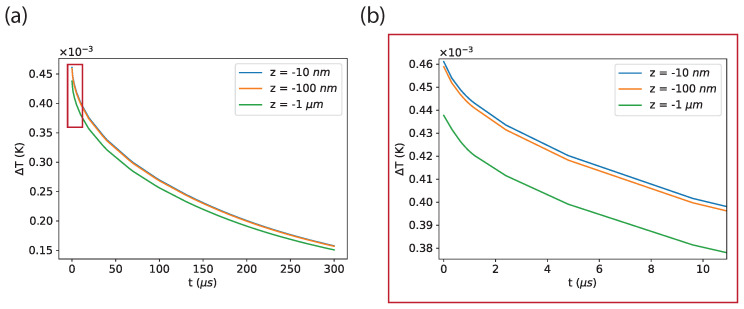
Results of the transient scenario for cell–water–diamond. This scenario starts, at t=0, with the temperature increase calculated in the previous stationary scenario. The temperature increases inside the diamond are plotted as a function of time for the points (r = 0 μm, z = −0.01 μm), (r = 0 μm, z = −0.1 μm), and (r = 0 μm, z = −1 μm) for the first 300 μs (**a**) and for the first 10 μs (**b**).

For the time-dependent case (see Figure 3), the equation considered is [44]:

(2) $$\rho c_{p}\frac{\partial T}{\partial t}+\nabla \cdot \left(-k\nabla T\right)=0,$$

where the same boundary conditions have been imposed as for the stationary scenario, and the initial state at t=0 has been set as the solution obtained from the stationary simulation.

The thermal parameters used for the different materials are reported in Table 1; we use the parameters reported in [45] for the cell and the ones present in the COMSOL [46] database for the other materials.

This time-dependent model has already been experimentally validated in previous studies [47], where the propagation of the heat deposited by a synchrotron X-ray nanobeam was investigated as a function of time, with a minimum time resolution of the order of one ps.

## 3. Results

As mentioned before, for glassy substrates the typical temperature difference $\Delta T_{glass}$, measured inside cells, ranges in the order of magnitude of a few Kelvins, as reported for different phenomena ranging from thermogenesis due to a mitochondrial uncoupler [9] to temperature variations related to an increase in synaptic activity [34]. Indeed, it is important to underline that, in Ref. [34], the cell adhered to a Petri dish, whereas, in our model, we consider the cell to be adhered to a bulk diamond, which has a much higher heat conductivity with respect to the Petri dishes; therefore, a much smaller temperature difference $\Delta T_{diam}$ is expected in this case.

In order to estimate $\Delta T_{diam}$, we first consider a cell adhered to a glass substrate surrounded by water (Figure 1a), and we calculate the dissipated heat power $P_{diss}$ that causes a temperature difference $\Delta T_{glass}=1$ K between the center of the cell and the boundaries of the glass substrate. Then, we consider a cell adhered to a bulk diamond surrounded by water (Figure 2a) in two different scenarios:

- The Stationary Scenario. In this scenario, the total heat power dissipated in the interior of the cell is the same value $P_{diss}$ as for the glassy substrate. In this case, we study the stationary temperature distribution both along the radial direction and in the vertical direction z inside the diamond. In particular, we calculate the temperature difference $\Delta T_{diam}$ between the center of the cell and the diamond substrate. This study gives us information about how close to the cell the sensor should be placed to measure the signal coming from a stationary phenomenon. Considering our recent results [34], we assume that the temporal duration of the phenomenon is 60 s.
- The Transient Scenario. In this scenario, at t=0, we impose the same spatial distribution of the temperature difference as the one calculated in the stationary scenario, and keep the same boundary conditions at 310.15 K as well. In this case, it is essential to determine the behavior of the temperature as a function of time at a certain depth z in the bulk diamond. This study provides information on how fast and how close to the cell the measurement should be carried out to resolve a very fast change in the cell temperature.

We considered a 300 μm thick diamond; this value is typical of commercial diamond substrates, and it corresponds to the thickness of a substrate that we used in a previous experiment [39]. Regarding the shape and size of the cell, it must be considered that both vary according to the cell’s function. The cell considered here has a hemispherical shape and is 40 μm in diameter because the eukaryotic cell size varies from 1 to 100 μm [48].

### 3.1. Estimation of the Total Dissipated Heat Power

The results regarding the estimation of the total dissipated heat power are represented in Figure 1. First, we considered the model in Figure 1a with the temperature of the glass and water fixed at 310.15 K (37.00 ${}^{\circ }$C) and with a fixed dissipated heat power density *Q* inside the cell. We then varied *Q* until we obtained a maximum stationary temperature difference of $\Delta T_{glass}=1$ K at the center of the cell. By using this procedure, we found a value of $P_{diss}=1.04\times 10^{-4}\;W$ after integration over the whole cell volume.

In all plots, we show the temperature increase with respect to the temperature of the boundaries. The temperature increase map is represented in Figure 1b, whereas the temperature increase profiles along the z-direction inside the cell/water and in the glass are represented in Figure 1c,d, respectively. The origin of the frame of reference is located at the center of the cell hemisphere. It can be noted that along the z-axis the temperature increase reaches a maximum at approximately 10 microns above the center of the cell and then decays on a spatial scale of a few tens of microns. Furthermore, the temperature increase at the cell–glass interface has already reduced by more than 0.3 K with respect to the value of its maximum (Figure 1c). The value of $P_{diss}$ found with this procedure is much greater than the value of $10^{-8}$ W attributed to the power consumption of the cell [49]. This is in accord with what was discussed in [12,13] and has already been mentioned in the Introduction of this paper.

### 3.2. Stationary Scenario for Diamond

Figure 2 presents the results regarding the stationary scenario. Additionally, in these plots, we consider the temperature increase with respect to the temperature of the boundaries. We calculate the temperature maps (Figure 2b) and the corresponding profiles along the vertical z-direction inside the cell, in the water (Figure 2c), and in the diamond (Figure 2d). We also calculate the temperature increase profile along the radial direction r at different depth values, z, inside the diamond (Figure 2e). The origin of the frame of reference is located at the center of the cell hemisphere. The most interesting outcomes are the following:

- The maximum temperature increase between the center of the cell and the bulk diamond $\Delta T_{diam}=0.63$ K is not so small compared to the one with the borosilicate $\Delta T_{glass}=1$ K.
- The temperature difference steeply decreases from its maximum towards the interface with the diamond, passing from ΔT(z=20μm) =0.6 K to ΔT(z=0μm) $=4.6\times 10^{-4}$ K. This means that more than 99.9% of the temperature increase $\Delta T_{diam}$ takes place inside the cell, which is out of the sensing region for sensors relying on bulk diamonds.
- The temperature difference between the cell/diamond interface and the boundaries is extremely small, about $4.6\times 10^{-4}$ K at most.

Further information can be gained by looking at the temperature profiles along the radial direction r. The temperature increase below the center of the cell and the boundaries is similar for the different depths, but, in all cases, it is extremely small, around $4.6\times 10^{-4}$ K. Moving away from the center of the cell, the temperature increase is reduced by a factor of two r=30μm and and by one order of magnitude at r=100μm.

### 3.3. Transient Scenario

The results regarding the transient scenario are presented in Figure 3. This scenario starts at t=0 with the spatial distribution of the temperature increase corresponding to the solution obtained from the stationary simulation. We calculate the temporal behavior of the temperature increases below the center of the cell for three depths: 0.01 μm, 0.1 μm, and 1 μm, for the first 300 μs (see Figure 3a). A zoom-in on the results corresponding to the first 10 microseconds is shown in Figure 3b. It can be noted that the temperature equilibrates to $\Delta T_{diam}=0$ on a time scale of hundreds of μs for each depth.

However, for the shallowest points, this process starts from a slightly higher temperature, corresponding to $\Delta T_{diam}=4.6\times 10^{-4}$ K for z = −0.01 μm and z = −0.1 μm, whereas $\Delta T_{diam}=4.4\times 10^{-4}$ K for z = −1 μm.

## 4. Discussion

Let us now discuss the feasibility of a temperature measurement for the two scenarios presented in the Results section. The discussion will be divided into the following steps:

- Description of the experimental procedure.
- Estimation of the sensitivity.
- Comparison of the estimated sensitivity with state-of-the-art sensitivities.

For the **stationary scenario**, the experimental procedure requires measurement of the temperature difference between two points: the one just below the center of the cell and the other outside the cell. This measurement could be carried out by using a gradiometric setup, e.g., a gradiometer exploiting Optically Detected Magnetic Resonance that employs two laser excitation beams focused on the two selected points.

The fluorescence emerging from the two positions should be collected simultaneously. Then, the temperature difference between the two spots can be calculated from the difference between the two fluorescence signals. The sensitivity η, in general, can be expressed as $\eta =\sigma \sqrt{\tau }$ where σ is the minimum detectable signal and τ is the temporal width chosen for the measurement. In order to be able to measure a signal of the mean value $\hat{s}$, it is necessary that $\hat{s}$ is greater than σ, so the minimal required sensitivity is $\eta_{req}=\hat{s}\sqrt{\tau }$. Obviously, a smaller value for the sensitivity is preferable. Let us consider Figure 2e: We measure at one point below the center of the cell and simultaneously at a distance r=60μm from the center of the cell, choosing as a reference z=−1μm. This leads to a signal $\hat{s}=3.6\times 10^{-4}$ K. Then, we set τ=60 s, in agreement with Ref. [34], where an increase in the temperature of 1 K is detected after the stimulation of the cell with a drug increasing the synaptic activity. Hence, we need a sensitivity of $\eta {}_{req}\left(-1\;\mu m\right)=2.8\times 10^{-3}\frac{K}{\sqrt{\mathrm{Hz}}}$. This value is two orders of magnitude smaller than the best sensitivity achieved in Petrini et al. (2020) [21], i.e., $\eta_{bio}=0.35\frac{K}{\sqrt{\mathrm{Hz}}}$ for intracellular temperature measurement with a biocompatible optical power of 1 mW.

For the **transient scenario**, the procedure would consist first of the measurement of the temperature just below the center of the cell in the absence of the transient phenomenon, i.e., 310.15 K, and then of the measurement of the temperature at the same point in the presence of the transient phenomenon. The last step of the procedure would be the evaluation of the difference between the temperatures in the two previous cases. For the shallowest points at z = −0.01 μm and z = −0.1 μm, we can estimate $\hat{s}=2.5\times 10^{-4}$ K, considering the arithmetic mean of the temperature difference profiles in Figure 3 and τ=300 μs. This corresponds to the required sensitivity of $\eta {}_{req}\left(-0.01\;\mu m\right)=4.3\times 10^{-6}\frac{K}{\sqrt{\mathrm{Hz}}}$. For z = −1 μm, we can estimate $\hat{s}=2.4\times 10^{-4}$ K and τ=300 μs, corresponding to a required sensitivity of $\eta {}_{req}\left(-1\;\mu m\right)=4.2\times 10^{-6}\frac{K}{\sqrt{\mathrm{Hz}}}$. These two values of sensitivity are very similar but, in this case as well, very far from the best sensitivity achieved in Ref. [21].

## 5. Conclusions

In summary, we have calculated the total dissipated heat power that creates a stationary increase of 1 K between the internal part of a cell and the surroundings, according to what was reported in a previous experiment, i.e., glass below the cell and water above. We then moved to another hypothetical environment composed of a diamond below the cell and water above. We used the calculated dissipated heat power to demonstrate that it is challenging to resolve both the transient phenomenon of a fast-changing temperature and the one where the temperature difference between the cell and the diamond is considered stationary.

It is worth mentioning that a related experimental study has been presented by Tanos et al. (2020) [50], where the focus was on the possibility of realizing wide-field thermal imaging with a bulk diamond, considering a two-dimensional heater that generates a fixed power. In their paper, the authors confirm, in the specific condition of their experiment, the general finding of our paper, i.e., that bulk diamonds cannot be exploited for sensing a local temperature increase because of their efficient thermalization properties. Specifically, we have focused our study on the relevant case of intracellular temperature measurements, whose expected experimental conditions are well known given our experimental results [34].

In our model, we have considered uniform heating inside the cell, but, from a biological point of view, it is more correct to consider a localized source of power dissipated in the correspondence of each mitochondrion, the organelle that generates most of the chemical energy needed by the cell. From this point of view, it would be interesting to extend the analysis conducted in the present paper by considering several sources of power dissipated in the interior of the cell, studying also the appearance of gradients inside the cell. Furthermore, in our model we consider a non-structured bulk diamond with a size of hundreds of micrometers, but nanostructured diamonds would probably perform better as temperature sensors. It is possible to fabricate single crystal diamond membranes with thickness values around 100 nanometers [51]: in this case, the smaller mass of the membrane and the reduced diamond cross-section area that are available for heat transfer out of the cell region should lead to a greater temperature difference between membrane regions beneath the cell and far away from it, even for the dissipated heat power treated in this paper. The arrays of diamond nanopillars should also show similar advantages compared to those of bulk diamonds: the reduced cross-section area of the pillars should limit the heat flux across the pillar bases towards the bulk diamond, leading to a smaller temperature difference between the cell and the location of the NV centers. 

## Figures and Tables

**Table 1:** Parameters used for the different materials in the model.

	*k* [W/(m·K)]	ρ [kg/m^3^]	$c_{p}$ [J/(kg·K)]
Diamond	2500	3515	516
Borosilicate glass	1.13	2230	754
Cell [45]	0.565	1050	3682
Water	0.62	994	4177

## Data Availability

The data that support the findings of this study are available from the corresponding author upon reasonable request.

## Supplementary Materials

The following supporting information can be downloaded at https://www.mdpi.com/article/10.3390/s24010200/s1: Mesh Sensitivity Analysis. Thermal Effects of Laser irradiation. Figures S1: Temperature at r=0 and at three different depths in the stationary scenario as a function of the different number of mesh elements. Figures S2: Temperature profiles along z and r directions obtained considering the heat generated by a 1 mW 532 nm laser [52].

## References

[1] Hille B. (1978). Ionic channels in excitable membranes. Current problems and biophysical approaches. Biophys. J..

[2] De Meis L., Ketzer L.A., Da Costa R.M., De Andrade I.R., Benchimol M. (2010). Fusion of the endoplasmic reticulum and mitochondrial outer membrane in rats brown adipose tissue: Activation of thermogenesis by Ca^2+^. PLoS ONE.

[3] Monti M., Brandt L., Ikomi-Kumm J., Olsson H. (1986). Microcalorimetric investigation of cell metabolism in tumour cells from patients with non-Hodgkin lymphoma (NHL). Scand. J. Haematol..

[4] Ghavami M., Rezaei M., Ejtehadi R., Lotfi M., Shokrgozar M.A., Abd Emamy B., Raush J., Mahmoudi M. (2013). Physiological temperature has a crucial role in amyloid beta in the absence and presence of hydrophobic and hydrophilic nanoparticles. ACS Chem. Neurosci..

[5] Prigogine I., Nicolis G. (1971). Biological order, structure and instabilities1. Q. Rev. Biophys..

[6] England J.L. (2013). Statistical physics of self-replication. J. Chem. Phys..

[7] Baiesi M., Maes C. (2018). Life efficiency does not always increase with the dissipation rate. J. Phys. Commun..

[8] Yang J.M., Yang H., Lin L. (2011). Quantum dot nano thermometers reveal heterogeneous local thermogenesis in living cells. ACS Nano.

[9] Fujiwara M., Sun S., Dohms A., Nishimura Y., Suto K., Takezawa Y., Oshimi K., Zhao L., Sadzak N., Umehara Y. (2020). Real-time nanodiamond thermometry probing in vivo thermogenic responses. Sci. Adv..

[10] Kiyonaka S., Kajimoto T., Sakaguchi R., Shinmi D., Omatsu-Kanbe M., Matsuura H., Imamura H., Yoshizaki T., Hamachi I., Morii T. (2013). Genetically encoded fluorescent thermosensors visualize subcellular thermoregulation in living cells. Nat. Methods.

[11] Chrétien D., Bénit P., Ha H.H., Keipert S., El-Khoury R., Chang Y.T., Jastroch M., Jacobs H.T., Rustin P., Rak M. (2018). Mitochondria are physiologically maintained at close to 50 C. PLoS Biol..

[12] Baffou G., Rigneault H., Marguet D., Jullien L. (2014). A critique of methods for temperature imaging in single cells. Nat. Methods.

[13] Macherel D., Haraux F., Guillou H., Bourgeois O. (2021). The conundrum of hot mitochondria. Biochim. Biophys. Acta (BBA)-Bioenerg..

[14] Suzuki M., Plakhotnik T. (2020). The challenge of intracellular temperature. Biophys. Rev..

[15] Sotoma S., Zhong C., Kah J.C.Y., Yamashita H., Plakhotnik T., Harada Y., Suzuki M. (2021). In situ measurements of intracellular thermal conductivity using heater-thermometer hybrid diamond nanosensors. Sci. Adv..

[16] Okabe K., Sakaguchi R., Shi B., Kiyonaka S. (2018). Intracellular thermometry with fluorescent sensors for thermal biology. Pflügers Arch.-Eur. J. Physiol..

[17] Li M., Chen T., Gooding J.J., Liu J. (2019). Review of carbon and graphene quantum dots for sensing. ACS Sens..

[18] Vetrone F., Naccache R., Zamarrón A., Juarranz de la Fuente A., Sanz-Rodríguez F., Martinez Maestro L., Martin Rodriguez E., Jaque D., Garcia Sole J., Capobianco J.A. (2010). Temperature sensing using fluorescent nanothermometers. ACS Nano.

[19] Kucsko G., Maurer P.C., Yao N.Y., Kubo M., Noh H.J., Lo P.K., Park H., Lukin M.D. (2013). Nanometre-scale thermometry in a living cell. Nature.

[20] Fujiwara M., Shikano Y. (2021). Diamond quantum thermometry: From foundations to applications. Nanotechnology.

[21] Petrini G., Moreva E., Bernardi E., Traina P., Tomagra G., Carabelli V., Degiovanni I.P., Genovese M. (2020). Is a quantum biosensing revolution approaching? Perspectives in NV-assisted current and thermal biosensing in living cells. Adv. Quantum Technol..

[22] Wu Y., Weil T. (2022). Recent developments of nanodiamond quantum sensors for biological applications. Adv. Sci..

[23] Gruber A., Drabenstedt A., Tietz C., Fleury L., Wrachtrup J., Borczyskowski C.v. (1997). Scanning confocal optical microscopy and magnetic resonance on single defect centers. Science.

[24] Bradac C., Gao W., Forneris J., Trusheim M.E., Aharonovich I. (2019). Quantum nanophotonics with group IV defects in diamond. Nat. Commun..

[25] Choi S., Agafonov V.N., Davydov V.A., Plakhotnik T. (2019). Ultrasensitive all-optical thermometry using nanodiamonds with a high concentration of silicon-vacancy centers and multiparametric data analysis. ACS Photonics.

[26] Nguyen C.T., Evans R.E., Sipahigil A., Bhaskar M.K., Sukachev D.D., Agafonov V.N., Davydov V.A., Kulikova L.F., Jelezko F., Lukin M.D. (2018). All-optical nanoscale thermometry with silicon-vacancy centers in diamond. Appl. Phys. Lett..

[27] Fan J.W., Cojocaru I., Becker J., Fedotov I.V., Alkahtani M.H.A., Alajlan A., Blakley S., Rezaee M., Lyamkina A., Palyanov Y.N. (2018). Germanium-vacancy color center in diamond as a temperature sensor. ACS Photonics.

[28] Alkahtani M., Cojocaru I., Liu X., Herzig T., Meijer J., Küpper J., Lühmann T., Akimov A.V., Hemmer P.R. (2018). Tin-vacancy in diamonds for luminescent thermometry. Appl. Phys. Lett..

[29] Bradac C., Lim S.F., Chang H.C., Aharonovich I. (2020). Optical nanoscale thermometry: From fundamental mechanisms to emerging practical applications. Adv. Opt. Mater..

[30] Guarina L., Calorio C., Gavello D., Moreva E., Traina P., Battiato A., Ditalia Tchernij S., Forneris J., Gai M., Picollo F. (2018). Nanodiamonds-induced effects on neuronal firing of mouse hippocampal microcircuits. Sci. Rep..

[31] Troise L., Hansen N.W., Olsson C., Webb J.L., Tomasevic L., Achard J., Brinza O., Staacke R., Kieschnick M., Meijer J. (2022). In vitro recording of muscle activity induced by high intensity laser optogenetic stimulation using a diamond quantum biosensor. AVS Quantum Sci..

[32] Jariwala D.H., Patel D., Wairkar S. (2020). Surface functionalization of nanodiamonds for biomedical applications. Mater. Sci. Eng. C.

[33] Romshin A.M., Zeeb V., Martyanov A.K., Kudryavtsev O.S., Pasternak D.G., Sedov V.S., Ralchenko V.G., Sinogeykin A.G., Vlasov I.I. (2021). A new approach to precise mapping of local temperature fields in submicrometer aqueous volumes. Sci. Rep..

[34] Petrini G., Tomagra G., Bernardi E., Moreva E., Traina P., Marcantoni A., Picollo F., Kvaková K., Cígler P., Degiovanni I.P. (2022). Nanodiamond–Quantum Sensors Reveal Temperature Variation Associated to Hippocampal Neurons Firing. Adv. Sci..

[35] Tisler J., Balasubramanian G., Naydenov B., Kolesov R., Grotz B., Reuter R., Boudou J.P., Curmi P.A., Sennour M., Thorel A. (2009). Fluorescence and spin properties of defects in single digit nanodiamonds. ACS Nano.

[36] Naydenov B., Dolde F., Hall L.T., Shin C., Fedder H., Hollenberg L.C., Jelezko F., Wrachtrup J. (2011). Dynamical decoupling of a single-electron spin at room temperature. Phys. Rev. B.

[37] Lindner S., Bommer A., Muzha A., Krueger A., Gines L., Mandal S., Williams O., Londero E., Gali A., Becher C. (2018). Strongly inhomogeneous distribution of spectral properties of silicon-vacancy color centers in nanodiamonds. New J. Phys..

[38] Golubewa L., Padrez Y., Malykhin S., Kulahava T., Shamova E., Timoshchenko I., Franckevicius M., Selskis A., Karpicz R., Obraztsov A. (2022). All-Optical Thermometry with NV and SiV Color Centers in Biocompatible Diamond Microneedles. Adv. Opt. Mater..

[39] Moreva E., Bernardi E., Traina P., Sosso A., Tchernij S.D., Forneris J., Picollo F., Brida G., Pastuović Ž., Degiovanni I. (2020). Practical applications of quantum sensing: A simple method to enhance the sensitivity of nitrogen-vacancy-based temperature sensors. Phys. Rev. Appl..

[40] Magaletti S., Mayer L., Le X.P., Debuisschert T. (2023). Magnetic sensitivity enhancement via polarimetric excitation and detection of an ensemble of NV centers. arXiv.

[41] Bernardi E., Moreva E., Traina P., Petrini G., Tchernij S.D., Forneris J., Pastuović Ž., Degiovanni I.P., Olivero P., Genovese M. (2020). A biocompatible technique for magnetic field sensing at (sub) cellular scale using Nitrogen-Vacancy centers. EPJ Quantum Technol..

[42] Losero E., Jagannath S., Pezzoli M., Goblot V., Babashah H., Lashuel H.A., Galland C., Quack N. (2023). Neuronal growth on high-aspect-ratio diamond nanopillar arrays for biosensing applications. Sci. Rep..

[43] McCloskey D.J., Dontschuk N., Broadway D.A., Nadarajah A., Stacey A., Tetienne J.P., Hollenberg L.C., Prawer S., Simpson D.A. (2020). Enhanced widefield quantum sensing with nitrogen-vacancy ensembles using diamond nanopillar arrays. ACS Appl. Mater. Interfaces.

[44] Incropera F.P., DeWitt D.P., Bergman T.L., Lavine A.S. (1996). Fundamentals of Heat and Mass Transfer.

[45] Mcintosh R.L., Anderson V. (2010). A comprehensive tissue properties database provided for the thermal assessment of a human at rest. Biophys. Rev. Lett..

[46] (2019). COMSOL Multiphysics^®^.

[47] Bonino V., Torsello D., Prestipino C., Mino L., Truccato M. (2020). Time and space resolved modelling of the heating induced by synchrotron X-ray nanobeams. J. Synchrotron Radiat..

[48] Mason K.A., Losos J.B., Singer S.R., Raven P.H., Johnson G.B. (2017). Biology.

[49] Loesberg C., van Miltenburg J., van Wuk R. (1982). Heat production of mammalian cells at different cell-cycle phases. J. Therm. Biol..

[50] Tanos R., Akhtar W., Monneret S., Favaro de Oliveira F., Seniutinas G., Munsch M., Maletinsky P., Le Gratiet L., Sagnes I., Dréau A. (2020). Optimal architecture for diamond-based wide-field thermal imaging. AIP Adv..

[51] Fairchild B.A., Olivero P., Rubanov S., Greentree A.D., Waldermann F., Taylor R.A., Walmsley I., Smith J.M., Huntington S., Gibson B.C. (2008). Fabrication of ultrathin single-crystal diamond membranes. Adv. Mater..

[52] Fraczek E., Savitski V.G., Dale M., Breeze B.G., Diggle P., Markham M., Bennett A., Dhillon H., Newton M.E., Kemp A.J. (2017). Laser spectroscopy of NV-and NV0 colour centres in synthetic diamond. Opt. Mater. Express.

