# Supplementary material for: Limitations of Bulk Diamond Sensors for Single-Cell Thermometry
Source: Sensors (Basel). 2023 Dec 29;24(1):200. doi: 10.3390/s24010200 (PMC10781228; doi:10.3390/s24010200)
Supplement: Supplementary file 1 [file sensors-24-00200-s001.zip › alessio_2023_supp_mat.pdf]

Limitations of bulk diamond sensors for single cell thermometry-Supplementary Material

Andrea Alessio [1], Ettore Bernardi [2*], Ekaterina Moreva [2], Ivo Pietro Degiovanni [2], Marco Genovese[2] and Marco Truccato[1].
[1]*Physics Department University of Turin, Via P. Giuria 1,10125 Turin, Italy and*
[2] *INRIM, Strada delle Cacce 91, 10135 Turin, Italy*

This document includes:

- Mesh Sensitivity Analysis
- Thermal Effects of Laser irradiation
- Figures S1 and S2

MESH SENSITVITY ANALYSIS

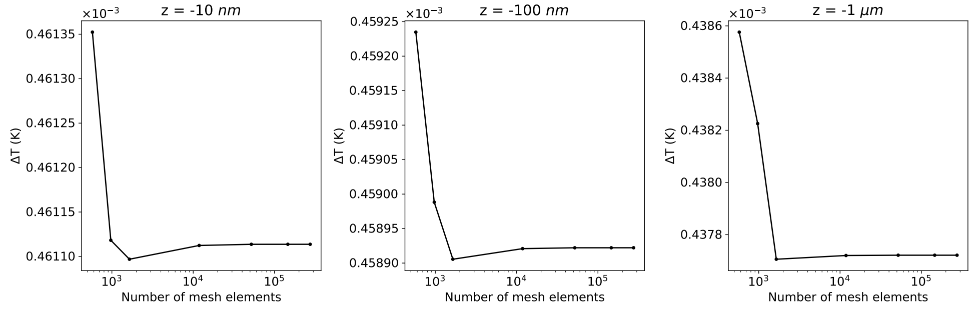

Figure S1: Temperature at $r = 0$ and at three different depths in the stationary scenario as a function of the different number of mesh elements. The largest number of elements was chosen in order to obtain element sizes comparable to the length scale under investigation.

In Figure S1, we have plotted the temperature at three different depths in the stationary scenario (specifically, the temperature values of Figure 2(e) at $r = 0$) as a function of the number of mesh elements. It can be noted that the results are stable already for a number of elements one order of magnitude smaller than the one chosen (274525). Such a large number of elements was chosen in order to obtain element sizes comparable to the length scale under investigation (10 nm).

EFFECT OF LASER IRRADIATION

Usually, to do temperature sensing with diamond color centers it is necessary to illuminate the sample with a laser beam. It is interesting to understand if the small gradient due the biological process is detectable upon the illumination by this laser beam. We consider the particular set-up used by our group in a previous experiment, see Ref. 39 of the main text. We consider a layer of NV centres with a thickness L=15 nm, at a depth d=10 nm below the diamond surface, with a density $N_{NV} = 3 * 10^{19}$ cm^{-3}. Considering a cross section $\sigma_{abs} = 3.1 * 10^{-17}$ cm^2 for the NV- center, see Ref. 52 of the main text, the absorbed fraction of the excitation power can be expressed as $f = 1 - \exp(-\sigma_{abs} N_{NV} L) \approx 10^{-3}$, resulting in a power absorbed around $P_{abs} = 1\,\mu$W.

We have performed numerical simulations considering as the heat source the dissipated power $P_{diss} = 1\,\mu$W with the shape of a cylinder 15 nm in thickness and $10\,\mu$m in diameter. Regarding the other parameters, the situation is identical to the one described in the main text of the paper under revision. The results are presented in figure S 2. We plotted the temperature profiles along z and r . The maximum increase in temperature is around 3.5*10-5 K, i.e. ten times smaller than the calculated signal of biological origin. Even if the temperature variation due to the application

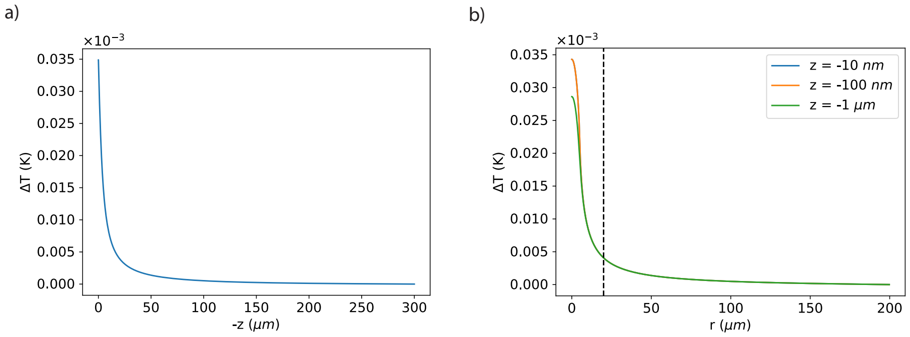

Figure S2: Temperature profiles along z (a) and r (b) directions obtained considering the heat generated by a 1 mW 532 nm laser (10 μm in diameter). The absorption from a 15 nm sensing layer at a depth of 10 nm is considered.

of the laser adds to the one of biological origin, the temperature gradient of biological origin is expected to be the dominant one since the perturbation induced by the laser is estimated being one order of magnitude lower than the target signal.
