# Supplementary figures and images for: Limitations of Bulk Diamond Sensors for Single-Cell Thermometry
Source: Sensors (Basel). 2023 Dec 29;24(1):200. doi: 10.3390/s24010200 (PMC10781228; doi:10.3390/s24010200)

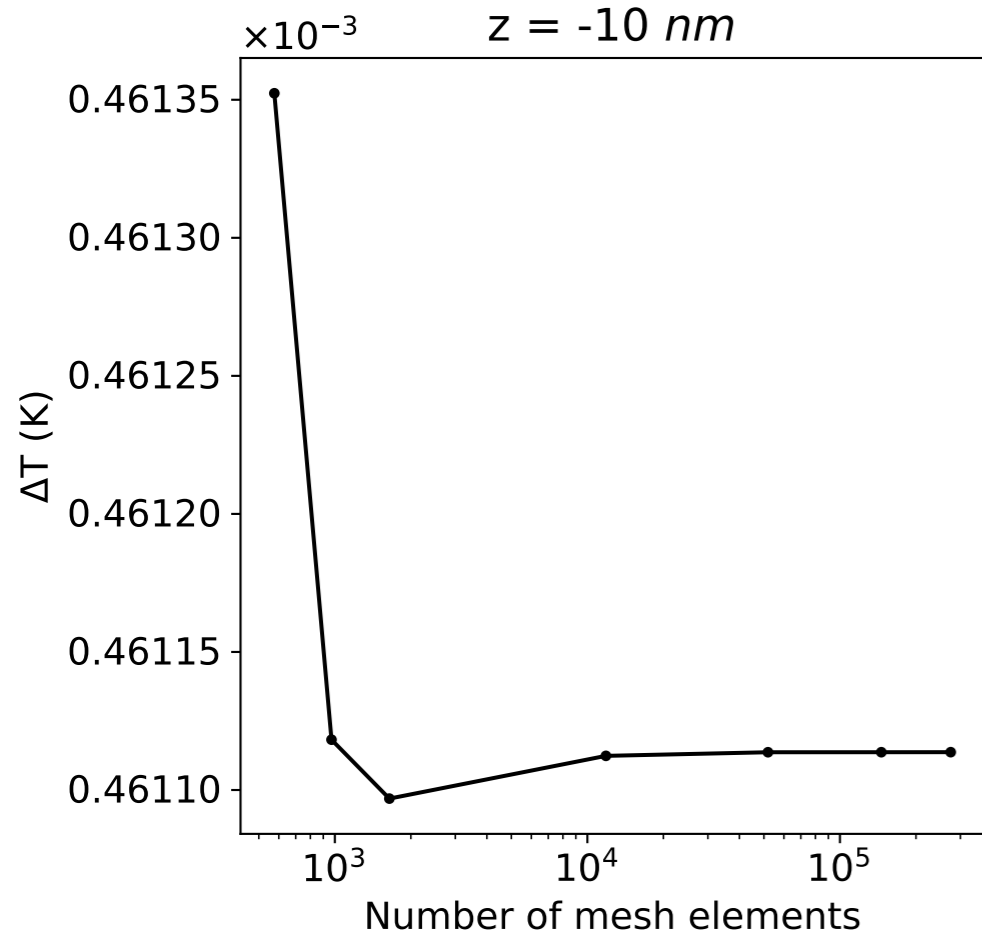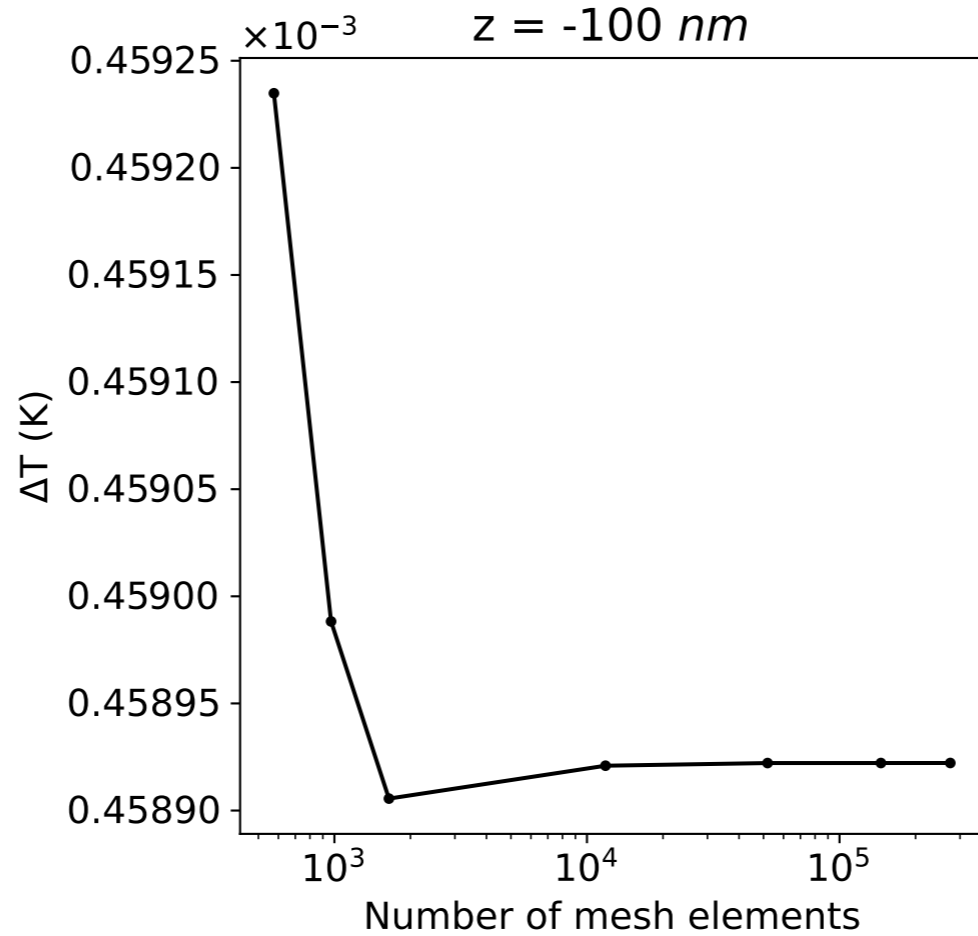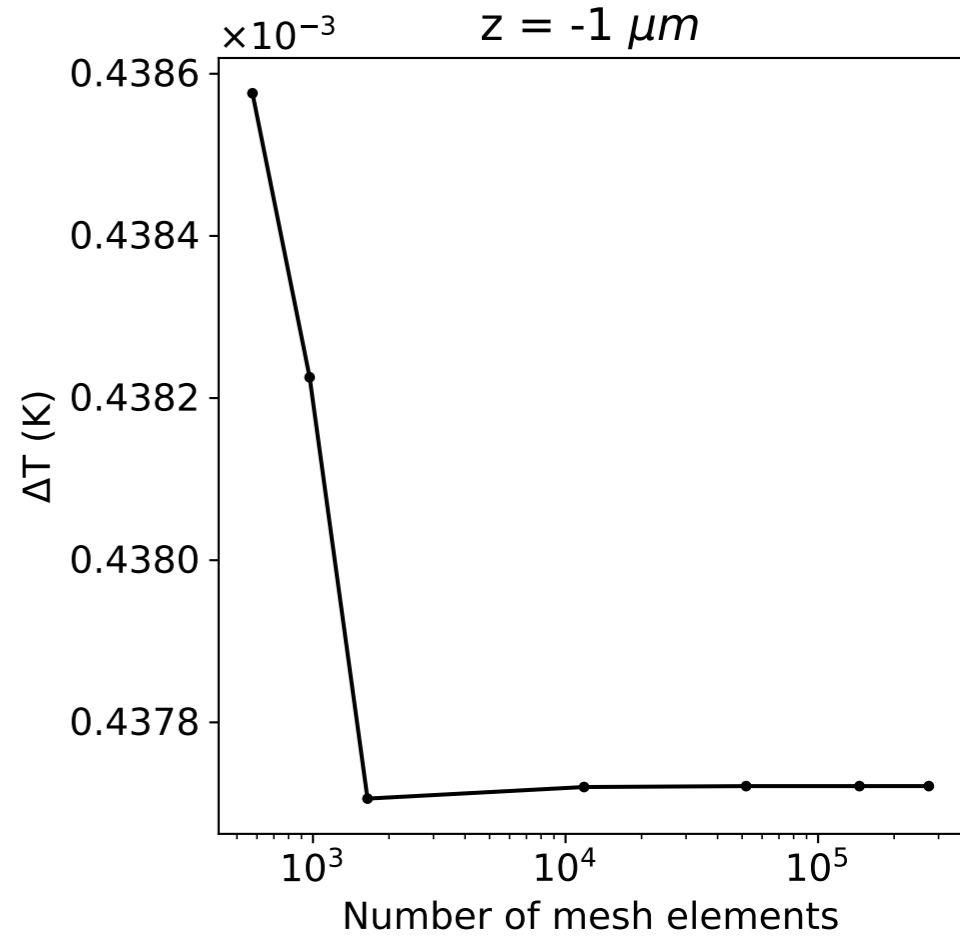

Supplement: Supplementary file 1 [file sensors-24-00200-s001.zip › Fig_S1.pdf]

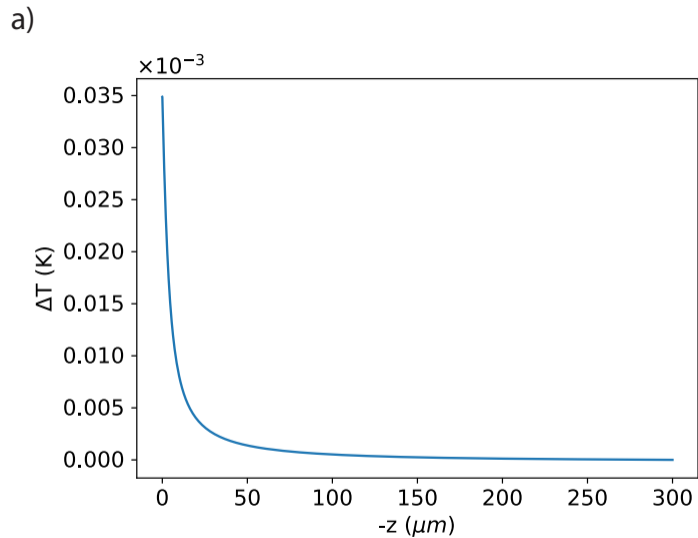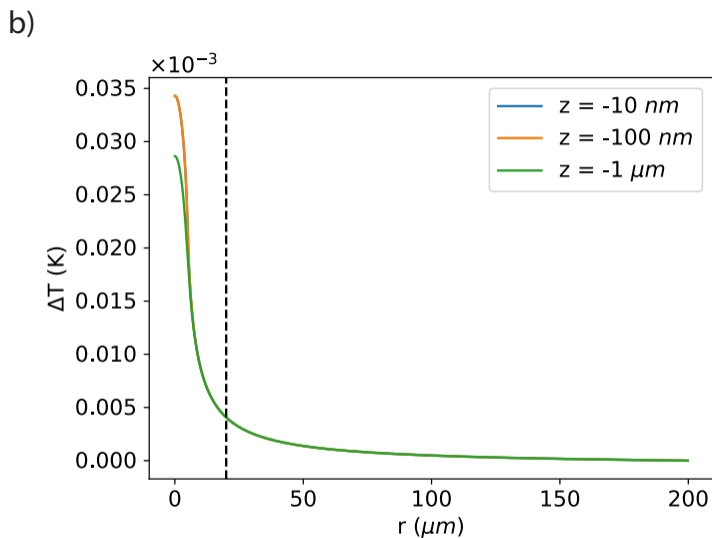

Supplement: Supplementary file 1 [file sensors-24-00200-s001.zip › Fig_S2.pdf]
